# Only α‐Gal bound to lipids, but not to proteins, is transported across enterocytes as an IgE‐reactive molecule that can induce effector cell activation

**DOI:** 10.1111/all.13873

**Published:** 2019-07-16

**Authors:** Patricia Román‐Carrasco, Barbara Lieder, Veronika Somoza, Marta Ponce, Zsolt Szépfalusi, Diana Martin, Wolfgang Hemmer, Ines Swoboda

**Affiliations:** ^1^ Molecular Biotechnology Section University of Applied Sciences Vienna Austria; ^2^ Department of Physiological Chemistry, Faculty of Chemistry University of Vienna Vienna Austria; ^3^ Department of Pediatrics and Adolescent Medicine Medical University of Vienna Vienna Austria; ^4^ Departamento de Producción y Caracterización de Nuevos Alimentos Instituto de Investigación en Ciencias de la Alimentación (CIAL) (CSIC‐UAM) Madrid Spain; ^5^ FAZ‐Floridsdorf Allergy Center Vienna Austria

**Keywords:** chylomicron, delayed allergic reaction, glycolipid, glycoprotein, red meat allergy, α‐Gal

## Abstract

**Background:**

The oligosaccharide galactose‐α‐1,3‐galactose (α‐Gal), present in mammalian proteins and lipids, causes an unusual delayed allergic reaction 3 to 6 hours after ingestion of mammalian meat in individuals with IgE antibodies against α‐Gal. To better understand the delayed onset of allergic symptoms and investigate whether protein‐bound or lipid‐bound α‐Gal causes these symptoms, we analyzed the capacity of α‐Gal conjugated proteins and lipids to cross a monolayer of intestinal cells.

**Methods:**

Extracts of proteins and lipids from beef were prepared, subjected to in vitro digestions, and added to Caco‐2 cells grown on permeable supports. The presence of α‐Gal in the basolateral medium was investigated by immunoblotting, thin‐layer chromatography with immunostaining and ELISA, and its allergenic activity was analyzed in a basophil activation test.

**Results:**

After addition of beef proteins to the apical side of Caco‐2 cells, α‐Gal containing peptides were not detected in the basolateral medium. Those peptides that crossed the Caco‐2 monolayer did not activate basophils from an α‐Gal allergic patient. Instead, when Caco‐2 cells were incubated with lipids extracted from beef, α‐Gal was detected in the basolateral medium. Furthermore, these α‐Gal lipids were able to activate the basophils of an α‐Gal allergic patient in a dose‐dependent manner.

**Conclusion:**

Only α‐Gal bound to lipids, but not to proteins, is able to cross the intestinal monolayer and trigger an allergic reaction. This suggests that the slower digestion and absorption of lipids might be responsible for the unusual delayed allergic reactions in α‐Gal allergic patients and identifies glycolipids as potential allergenic molecules**.**

## Graphical abstract

α‐Gal is present in both lipids and proteins from beef, but only when it is bound to beef lipids, the oligosaccharide can cross the Caco‐2 cell monolayer. α‐Gal carried by lipids is packaged by the enterocytes into chylomicrons, which are then able to activate the basophils of an α‐Gal allergic patient. The slower in vivo digestion and absorption of lipids explain the delayed allergic reactions to α‐Gal. α‐Gal: galactose‐ α‐1,3‐galactose

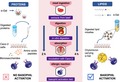

**Abbreviations**
AP: apical
ApoB: apolipoprotein B
BAT: basophil activation test
BL: basolateral
BSA: bovine serum albumin
CCD: cross‐reactive carbohydrate determinants
DMEM: Dulbecco's modified Eagle's medium
FBS: fetal bovine serum
HSA: human serum albumin
mAb: monoclonal antibody
PBS: phosphate buffer saline
RT: room temperature
TLC: thin‐layer chromatography
α‐Gal: galactose‐α‐1,3‐galactose

## 1 INTRODUCTION

The existence of IgE antibodies directed against glycans on plant and invertebrate proteins, termed cross‐reactive carbohydrate determinants (CCDs), has been known for a long time,^1^ but the presence of these antibodies in patients' sera seems to lack clinical relevance.^2^ However, just a decade ago, it was shown that IgE antibodies to another glycan, the oligosaccharide galactose‐α‐1,3‐galactose (α‐Gal), can cause immediate anaphylactic reactions to the monoclonal anti‐cancer antibody cetuximab^3^ and trigger a new delayed form of food‐induced anaphylaxis, in which the onset of symptoms, unlike in protein‐based food allergies, occurs 3 to 6 hours after the consumption of mammalian meat.^4^, ^5^

The mechanisms leading to the differences in the response to CCDs and α‐Gal are still elusive. Furthermore, the reason for the late onset of symptoms in case of α‐Gal allergy to mammalian meat is not understood. It has been suggested that the delay in symptoms might be due to a delay in the occurrence of the allergenic molecules in the circulation, meaning that the different digestion and transportation of the meat nutrients might be responsible for the late symptoms.^5^ The α‐Gal carbohydrate structure is not only present on mammalian glycoproteins, but also on glycolipids. Whereas peak levels of amino acids and small peptides, the digestion products of orally ingested proteins, are detected in the blood 1‐2 hours postmeal^6^, ^7^, ^8^, it takes 4‐5 hours for dietary lipids to reach the circulation.^9^

The majority of ingested proteins is digested into small peptides and single amino acids by a number of peptidases in the stomach and the small intestine. Single amino acids, di‐, and tripeptides are taken up by enterocytes. Di‐ and tripeptides are then further hydrolyzed by cytosolic peptidases, and amino acids are finally transported to the bloodstream. In order to trigger an IgE‐mediated allergic reaction, food allergens require certain characteristics that ensure resistance to proteolytic degradation as well as intact transportation through the gut epithelium by endocytosis. Furthermore, to activate mast cells or basophils and elicit an allergic reaction, a food allergen needs to cross‐link IgE antibodies on the surface of the effector cells. So far, nothing is known about the stability of α‐Gal carrying glycoproteins. However, to cause IgE cross‐linking and subsequent activation of effector cells, meat proteins or peptides would need to carry more than one α‐Gal epitope after crossing the intestinal epithelium.

Dietary lipid digestion and adsorption, on the other hand, are a more complex process. Large, insoluble lipid (predominantly triglycerides) aggregates are first broken down physically into small droplets. Bile salts and phospholipids form a layer coating the hydrophobic molecules to promote the emulsification and solubilization of lipids in the aqueous medium, forming a micelle.^10^ Then, pancreatic lipase hydrolyzes the lipids inside the micelle into free fatty acids, di‐, and monoglycerides. The micelles carry the free fatty acids and monoglycerides to the surface of enterocytes, where they get absorbed. In the enterocytes, the fatty acids and monoglycerides are converted again into triglycerides, which are then packaged together with phospholipids, cholesterol esters, and apolipoprotein B‐48 into lipoprotein particles, called chylomicrons.^11^ Chylomicrons leave the enterocytes by exocytosis and are released into the lymphatic system, finally entering the bloodstream via the thoracic duct roughly 4 hours after a meal ingestion.^12^ It is known that α‐Gal is predominantly linked to glycosphingolipids, which represent a diverse group of membrane‐bound glycolipids with several different biological functions.^13^ Little is known about the resistance to digestive enzymes of glycosphingolipids containing α‐Gal. Theoretically, glycosphingolipids binding α‐Gal, similarly to phospholipids, could be incorporated into lipid micelles. Despite the lack of information about the particular absorption mechanism of lipids containing α‐Gal, it has been suggested that they could be also incorporated in the surface of chylomicrons, reaching the systemic circulation several hours after ingestion.^3^, ^13^, ^14^ This mechanism would explain the delayed reactions after ingesting α‐Gal containing meat.

The aim of this study was to investigate whether α‐Gal bound to beef proteins or lipids would be transported across the intestinal epithelial cells and cause the activation of the basophils from an α‐Gal allergic patient. Caco‐2 cell monolayers incubated with in vitro digested allergens have been previously used to study the transport of allergenic proteins through the intestinal epithelium.^15^ In vitro digestions of both lipid and protein extracts of beef were performed. The digested molecules were then added to the apical side of Caco‐2 cell monolayers cultured on permeable supports, and the basolateral media were analyzed for the presence of α‐Gal and the capability to cause the activation of basophils of an α‐Gal allergic patient.

## 2 METHODS

### 2.1 Meat extracts

For protein extract preparation, 5 g of grilled beef and chicken were homogenized by freezing the samples in liquid nitrogen and subsequently grinding them to a fine powder using a mortar and a pestle. The tissue powder was suspended in 50 mL phosphate buffer saline (PBS) pH 7.5 and incubated overnight at 4°C on a rocking platform. After centrifugation at 4500 *g* for 30 minutes at 4°C, the supernatant was collected, the extracts were freeze‐dried, and the protein concentration was determined by Bradford (Bio‐Rad Laboratories).

Lipids were extracted from grilled beef and chicken meat as described by Smith and Prieto.^16^ In brief, a piece of meat was cut into small pieces and homogenized with water. To 3 volumes of aqueous solution, 8 volumes of methanol followed by 4 volumes of chloroform were added. The mixture was sonicated, incubated for 20 minutes at 25°C, and then centrifuged for 20 minutes at 9000 *g* (at 25°C) using Nalgene^®^ Oak Ridge Centrifuge Tubes, Teflon^®^ FEP from Thermo Fisher Scientific. The supernatant was collected, and the pellet extracted once more as described above. The supernatants were combined, and the pellet was further extracted, first with 1:1 chloroform/methanol (v/v) followed by extraction with a 2:1 mixture of chloroform/methanol (v/v) and centrifugation as described above. The collected supernatants were combined and centrifuged at 9000 *g* (25°C) to remove any particles and then dried in a rotary evaporator. The dried lipids were recovered in 2:1 chloroform/methanol (v/v) and stored at −20°C.

### 2.2 In vitro digestion of meat extracts

The simulated gastrointestinal digestion of the extracted meat proteins was carried out following the method described by Moreno et al.^17^ Lyophilized powder from protein extracts containing 35 mg of protein was dissolved in simulated saliva fluid (potassium phosphate 0.005 mol/L, CaCl_2_ 0.004 mol/L, NaCl 0.04%, pH 6.5) at 37°C. Before the pH was adjusted to 2.9 with HCl (5 mol/L), an aliquot representing the oral phase was taken. After the addition of pepsin (Sigma‐Aldrich) at a physiological enzyme to substrate ratio of 182 U/mg of protein, the gastric digestion was performed by incubating the mixture with agitation at 37°C. Aliquots were taken after 5 and 60 minutes of gastric digestion, and pepsin was irreversibly inactivated in the aliquots by increasing the pH to 7.5 with 1 mol/L NaHCO_3_. To the rest of the digest, Bis‐Tris (0.25 mol/L, pH 6.5) and CaCl_2 _(7.6 mmol/L final concentration) were added and the pH was adjusted to 7 with 1 mol/L NaHCO_3_ to perform the simulated duodenal digestion. For this, pancreatic bovine trypsin (EC 232‐650‐8, type I 10 100 BAEE U/mg protein, Sigma‐Aldrich) and pancreatic bovine α‐chymotrypsin (EC 232‐671‐2; type I‐S; 55 U/mg protein, Sigma‐Aldrich) were added to the duodenal mix at enzyme to substrate ratios of 34.5 U/mg protein and 0.44 U/mg protein, respectively.^17^ The mixture was incubated at 37°C while shaking (150 rpm). Aliquots were taken 2, 30, 60, and 90 minutes after the addition of trypsin. The digestion was always stopped by adding Pefabloc^®^ SC (Sigma‐Aldrich) to a final concentration of 5 mmol/L to each aliquot.

In vitro digestion of lipids was performed according to the method described by Martin et al.^18^ Either 600 mg or 450 mg or 300 mg of extracted beef or 600 mg of extracted chicken lipids in 2:1 chloroform/methanol (v/v) was transferred to a glass vial and dried under a nitrogen stream. Then, 6 mL of Trizma‐maleate buffer (0.1 mol/L, pH 7.5), prewarmed to 37°C, was added to the vial containing the dried lipids. Bile secretion was mimicked by addition of a mixture of bile salts (10 mmol/L in final volume of 10 mL), lecithin (3 mmol/L), CaCl2 (12.5 mmol/L), and NaCl (0.5 mol/L) in 4 mL of Trizma‐maleate buffer that had been prewarmed to 37°C and homogenized by sonication. The whole mixture was further emulsified by sonication, and simulation of intestinal digestion started with the addition of 1 mL of pancreatin extract (0.3 g of 8×USP pancreatin from Sigma‐Aldrich in Trizma‐maleate buffer, stirred for 10 minutes, centrifuged at 1600 *g* for 15 minutes). Digestion was carried out at 37°C while shaking (150 rpm) for 1 hour. Then, the digests were centrifuged (4000 *g*, 20°C, 40 minutes) obtaining a precipitate of enzymes, an upper oily phase and a central micellar phase that contained the digested lipids forming micelles together with the bile salts and the phosphatidylcholine. This micellar phase was used in further experiments.

### 2.3 Intestinal transport experiments using Caco‐2 cells

Culture conditions and cell viability assay details of Caco‐2 (human colorectal cancer) cells are described in the Methods S1. Intestinal transport experiments were performed 21 days after seeding of the cells. For all the uptake experiments, Caco‐2 cells were incubated with low‐glucose Dulbecco's modified Eagle's medium (DMEM) without addition of FBS to the basolateral compartment. Undigested or digested lipid extracts were diluted in low‐glucose DMEM containing 0.1% FBS and added to the apical compartment of the Caco‐2 cells, which were then incubated for 1, 2, 4 hours, and/or overnight at 37°C. Undigested and digested protein extracts were diluted in low‐glucose DMEM containing 0.1% FBS and were always applied in a concentration of 1 mg/mL, and cells were incubated with the protein extracts overnight at 37°C. The undigested beef lipid extracts were emulsified by addition of 2 mmol/L sodium taurocholate and 1.5 mmol/L L‐α‐Phosphatidylcholine^19^ and were also applied in a concentration of 1 mg/mL. For addition of the micellar phase of digested lipids, nontoxic concentrations of the micellar phase first had to be determined in cell viability (MTT) assays (see Methods S1). This was necessary because bile salts in high concentrations are known to have a toxic effect on cells. Based on the results of the MTT assays, the micellar phases were applied in a dilution of 1:60. Incubations were performed with different concentrations of digested beef lipids (1.0, 0.75, and 0.5 mg/mL). As negative controls, digested chicken lipids (1 mg/mL) were applied to the cells, since chicken does not express α‐Gal, or only medium.

For analysis of the proteins present in the apical medium applied to the cells and in the basolateral medium of the exposed Caco‐2 cells, SDS‐PAGEs and immunoblots were carried out, and to analyze the lipids, thin‐layer chromatography experiments were performed. Furthermore, to analyze whether chylomicrons had been formed in the Caco‐2 cells after addition of undigested or digested lipid extracts, the expression of the chylomicron marker apolipoprotein B‐48 (ApoB‐48) was investigated by immunoblotting using an anti‐ApoB antibody (see Methods S1).^19^, ^20^

### 2.4 SDS‐PAGE and immunoblot

SDS‐PAGEs of beef and chicken proteins, digested proteins applied to the apical side of Caco‐2 cells and of the collected basolateral media as well as anti‐α‐Gal immunoblots are described in the Methods S1.

### 2.5 Thin‐layer chromatography and thin‐layer chromatography immunostaining

Undigested and in vitro digested beef and chicken lipids were spotted on aluminum‐backed thin‐layer chromatography (TLC) plates of Silica Gel 60 (Merck Millipore). For TLC analysis of lipid digestion products, a partition of the lipids was carried out following the method by Folch et al.^21^ Digestion products or basolateral media were first dried in a vacuum centrifugal evaporator, and then, the pellets were extracted with chloroform/methanol 2:1 (v/v) and washed with 0.2 volumes of water. After centrifugation (13 000 *g*, 1 minute), an upper phase containing polar molecules and a lower phase containing apolar molecules were obtained. The phases were collected, evaporated, re‐suspended in chloroform/methanol 2:1 (v/v), and spotted on TLC plates. TLC was performed using chloroform/methanol/water (60:35:8) for separation of polar lipids or hexane/diethyl ether/acetic acid (75:25:1.5) for separation of apolar lipids as mobile phases. After drying, the plates were reversibly stained with iodine vapors by exposing them to I_2_ crystals (Sigma‐Aldrich) in a closed jar at room temperature for about 5 minutes. For immunostaining, TLC plates were dried and then soaked in a solution of 0.2% poly‐isobutyl‐methacrylate (Sigma‐Aldrich) in hexane for 1 minute. The dried, plastic‐coated plates were blocked for 60 minutes in PBS containing 0.1% human serum albumin (HSA) at room temperature (RT) and rinsed three times with PBS. Then, they were incubated either with an anti‐α‐Gal antibody (m86 mAb, Absolute Antibody) coupled to horseradish peroxidase (HRP) (1:1000 in PBS, 90 minutes, RT) or with patient's serum (1:10 in PBS, 2 hours, RT) followed by exposure to a HRP‐coupled mouse anti‐human IgE antibody (1:5000 in PBS, 60 minutes; Southern Biotech). Plates were finally incubated with HRP substrate (SuperSignal™ West Pico, Thermo Fisher Scientific) and detected using the FluorChem^®^, Protein Simple device (Biozyme Scientific GmbH).

### 2.6 α‐Gal sandwich ELISA

To analyze whether α‐Gal detected in the basolateral chamber medium of Caco‐2 cells that had been exposed to lipids was attached to chylomicrons, an α‐Gal sandwich ELISA was developed. For this, wells of a 96‐well polystyrene microtiter plate (Maxisorp Nunc) were coated with 100 µL of the anti‐α‐Gal antibody (m86 mAb, Absolute Antibody) diluted 1:500 in bicarbonate buffer (pH 9.6) and incubated overnight at 4°C. After washing with PBS, wells were blocked with 0.1% HSA in PBS for 2.5 hours at 37°C. According to the method described by Cartwright et al,^22^ the basolateral chamber medium from Caco‐2 cells incubated with lipid mixtures was first centrifuged for 20 minutes at 13 000 *g* (at 16°C) to induce floating of the chylomicrons to the surface. The top 100 µL of each tube was collected, diluted 1:2 or 1:4 with PBS or was not diluted, and was added to the wells of the ELISA plate. After incubation for 15 minutes at 37°C and further for 2 hours at room temperature on a rocking platform, the wells were washed three times with PBS, followed by an incubation with a HRP‐labeled goat anti‐apolipoprotein B polyclonal antibody (diluted 1:2500 in PBS; ab20047, Abcam) for 1 hour at RT. Afterward, the wells were thoroughly washed with PBS and 100 µL of ABTS‐peroxidase substrate solution (1 mg/mL) was applied to each well, before the absorbance was measured at 405 nm. All samples were analyzed in the ELISA in triplicates. The data obtained in the ELISA were analyzed using one‐way ANOVA with multiple comparison tests in GraphPad Prism 6 (GraphPad Software).

### 2.7 Basophil activation tests

Basophil activation tests (BATs) are described in the Methods S1.

## 3 RESULTS

### 3.1 α‐Gal is bound to glycoproteins and glycolipids of beef

**Figure 1:**
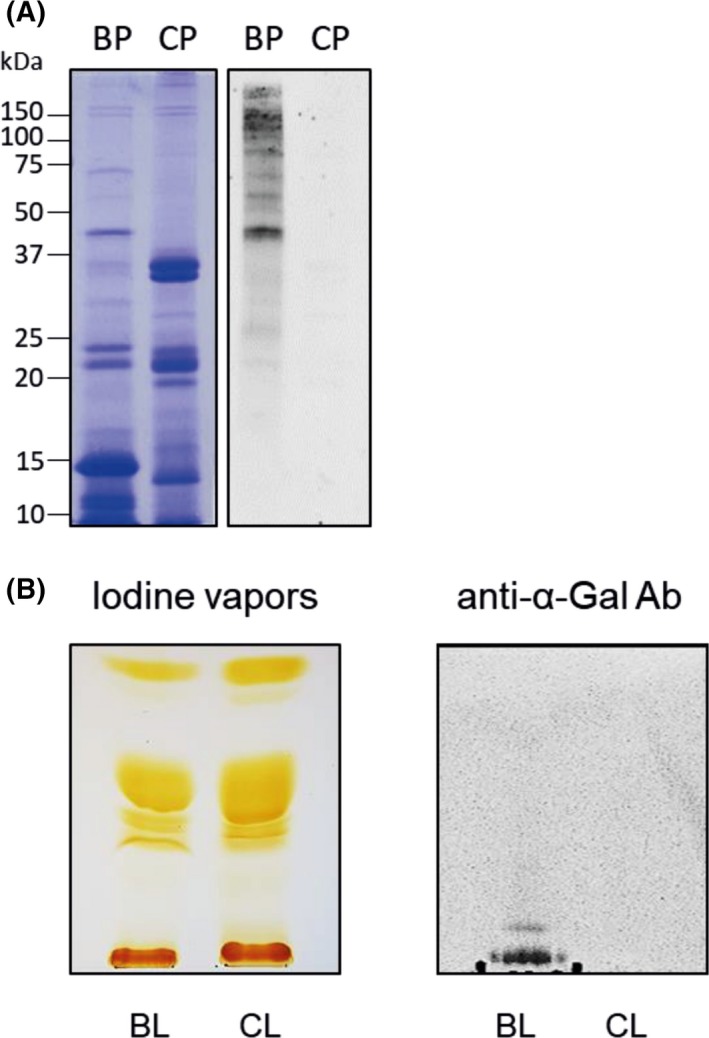
α‐Gal is present in beef glycoproteins and glycolipids. A, Coomassie‐stained 15% SDS‐PAGE (left) and anti‐α‐Gal immunoblot (right) of beef (BP) and chicken (CP) protein extracts. Molecular weights are indicated in the margin. B, Thin‐layer chromatography of beef (BL) and chicken (CL) lipid extracts, stained with iodine vapors (left) and immunostained with an anti‐α‐Gal antibody (Ab) (right). Chloroform/methanol/water (60:35:8) was used as a mobile phase

To investigate whether α‐Gal was present on both beef glycoproteins and glycolipids, proteins and lipids were extracted from grilled beef. The average extraction yield of water‐soluble proteins was around 0.5%, and the extraction yield of lipids was around 10%. The presence of α‐Gal on beef proteins was evaluated by immunoblotting, and its presence on lipids was analyzed by thin‐layer chromatography immunostaining. For control purposes, protein and lipid extracts from grilled chicken were also prepared and evaluated for the presence of α‐Gal, because it is known that birds do not express α‐Gal.^23^ Coomassie staining of beef and chicken proteins separated by SDS‐PAGE showed the overall integrity of the proteins, but also differences in the banding pattern (Figure 1A). A specific anti‐α‐Gal antibody detected several α‐Gal carrying proteins in immunoblots in the beef protein extract, whereas no binding to α‐Gal was observed in the chicken protein extract (Figure 1A). Most α‐Gal carrying proteins in the beef extract were of higher molecular weight (above 45 kDa), which is in accordance with previous studies demonstrating that α‐Gal carrying proteins are of relatively high molecular weight.^24^ Beef and chicken lipid extracts were separated by thin‐layer chromatography (TLC). Iodine staining of the TLC plate showed similar migration patterns of beef and chicken lipids. Whereas apolar lipids migrate to the top of the plate, the more polar ones would remain closer to the baseline (Figure 1B). Interestingly, immunostaining of the TLC plate with the anti‐α‐Gal specific antibody showed that α‐Gal was present on beef lipids, whereas no binding of the antibody occurred to the chicken lipid extract (Figure 1B). Apparently, glycolipids carrying α‐Gal in the beef lipid extract have several bound sugar moieties. This makes them more polar, and therefore, they remain at the origin of the TLC plate, similar to phospholipids.

### 3.2 α‐Gal is not detected in the basolateral media of Caco‐2 cells incubated with digested proteins

**Figure 2:**
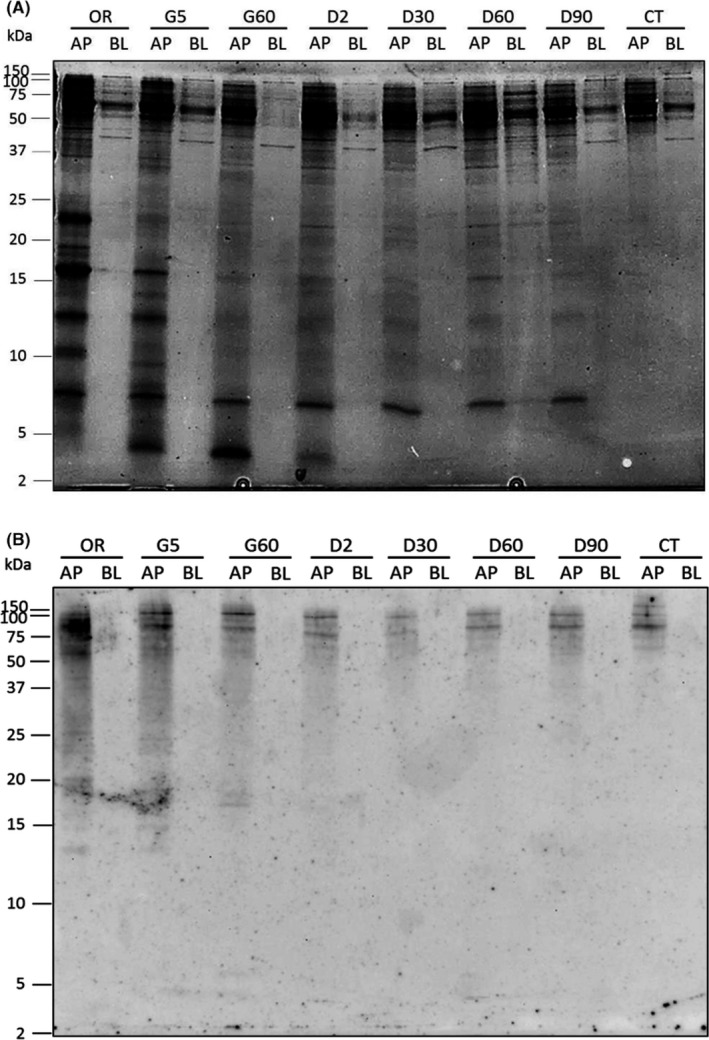
α‐Gal is not detected in the basolateral media of Caco‐2 cells incubated with undigested and digested proteins. A, Coomassie‐stained 16.5% Tris‐Tricine gel and B, anti‐α‐Gal immunoblot of undigested and in vitro digested beef proteins applied to the apical side (AP) and collected from the basolateral medium (BL) of Caco‐2 cells cultured on permeable supports. Cells were incubated with undigested proteins (OR), or with proteins exposed to 5 min and 60 min of peptic digestion (G5 and G60), and to 2 min, 30 min, 60 min, and 90 min of duodenal digestion (D2, D30, D60, and D90). For control purposes, cells were also incubated with DMEM containing 0.1% of FBS (CT). In the left margin, the sizes of molecular weight marker are shown in kDa

To evaluate whether protein‐bound α‐Gal is transported across the intestinal epithelial cells, a tight monolayer (TEER > 400 Ω/cm^2^) of differentiated Caco‐2 cells was cultured on permeable supports. To mimic processes in the gastrointestinal tract as close as possible, beef protein extracts were first digested in vitro according to the method from Moreno et al.^17^ Aliquots were taken from the oral phase (OR in Figure 2), at different times of gastric (5 minutes = G5, 60 minutes = G60) and duodenal digestion (2 minutes = D2, 30 minutes = D30, 60 minutes = D60 and 90 minutes = D90), and were applied on the apical (AP) side of the Caco‐2 cell monolayer. For control purposes, only DMEM containing 0.1% FBS was added to AP side of the cells (CT). Samples applied to the apical side were analyzed on a Coomassie‐stained Tris‐Tricine gel (lanes AP in Figure 2A). As can be seen in Figure 2A, the overall intensity and protein band pattern changed during the digestion process. Major bands visible in the oral phase (OR), which represents the undigested sample, had molecular weights of 7, 10, 12, 16, 23, and 50‐150 kDa. Protein bands of 10 and 23 kDa became already fainter after 5 minutes of gastric digestion (G5), and after 60 minutes of gastric digestion (G60), the majority of the strong bands were no longer visible. Only the protein band at 7 kDa and protein bands between 50 and 70 kDa remained largely the same even after 90 minutes of duodenal digestion. A novel protein band of 4 kDa appeared after 5 minutes of gastric digestion (G5), remained over the gastric digestion process, but turned fainter already 2 minutes after duodenal digestion (D2) and then disappeared.

After overnight exposure of the Caco‐2 cells to the undigested and digested beef proteins, the basolateral media were collected and also analyzed on Coomassie‐stained Tris‐Tricine gels. In the lanes BL of Figure 2, samples taken after overnight exposure of the Caco‐2 cells are shown. As can be seen in Figure 2A, hardly any protein bands and none of the strong bands observed in the apical media (AP) were present in the basolateral media (BL) of cells exposed to undigested and digested proteins. Only small amounts of proteins of 16, 25, 40, and 50‐150 kDa could be observed in the basolateral media, indicating that the majority of proteins were not transferred intact through the Caco‐2 cells. The only strong band visible in the basolateral media was a band of 70 kDa, which was also present in the apical media. Immunoblots carried out with an anti‐serum albumin antibody showed that the 70 kDa was bovine serum albumin (BSA) (Figure S1) which is described to be absorbed intact in the small intestine and transported as such into the lymph or blood.^25^ Because 0.1% of FBS (containing serum albumin) was added to the media applied to the apical side of the cells, BSA was also present in the experiments performed only with medium (CT in Figure S1).

The presence of protein‐bound α‐Gal in the AP and BL media was analyzed by immunoblotting using the anti‐α‐Gal antibody (Figure 2B). In the AP samples, α‐Gal carried by beef proteins was strongly detected in the oral phase sample (OR in Figure 2B), representing undigested beef proteins, at molecular weights above 50 kDa. In the samples taken of the protein digests, the signal intensity gradually decreased, indicating that the α‐Gal carrying proteins were progressively degraded. Only two protein bands of molecular weights of ~75 kDa and ~90 kDa were still detected in the AP samples after 90 minutes of gastric digestion (D90). A smear, typical for glycoproteins separated by SDS‐PAGE, 26 can be seen in all AP samples in the α‐Gal immunoblot (Figure 2B). The anti‐α‐Gal antibody also recognized glycoproteins in the AP medium of the Caco‐2 cells incubated with medium (CT). This is not surprising, since FBS added to medium would also contain protein‐bound α‐Gal. However, Figure 2B also shows that α‐Gal was not present in any of the basolateral media, indicating that proteins carrying α‐Gal were not transported through the Caco‐2 cell monolayer to the basolateral medium.

### 3.3 α‐Gal is present in the basolateral medium of Caco‐2 cells incubated with digested beef lipids

**Figure 3:**
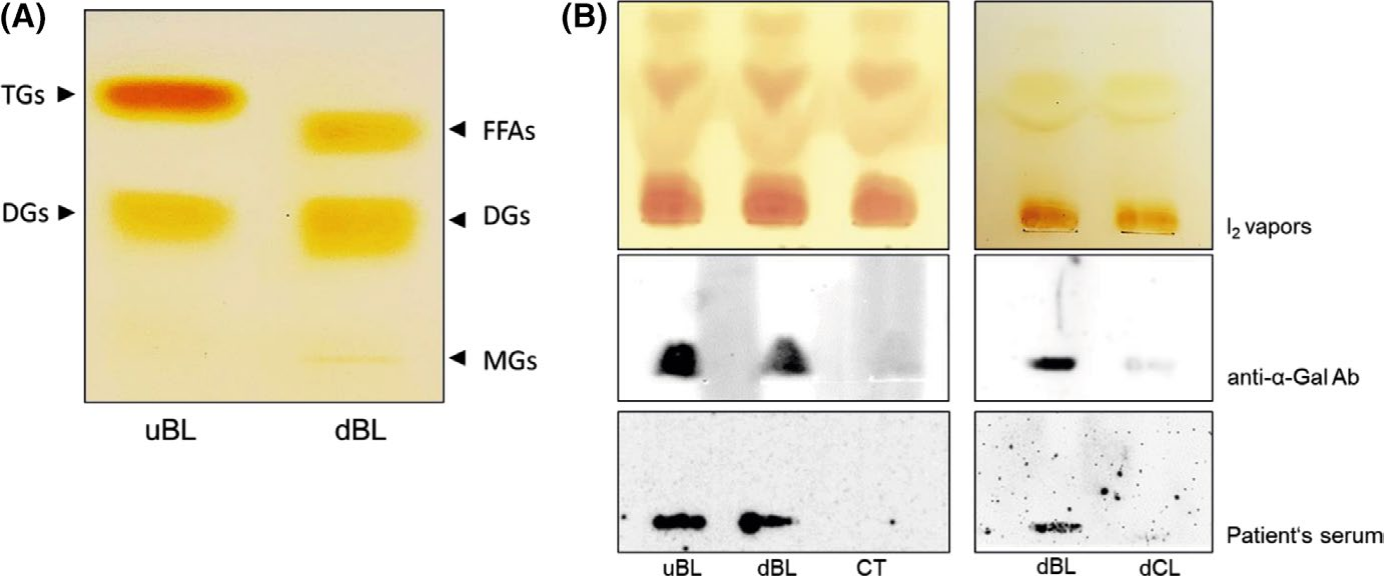
α‐Gal is detected in the basolateral medium of Caco‐2 cells incubated with digested beef lipids. A, Lower phases of undigested (uBL) and digested (dBL) beef lipids extracted by Folch were separated on a TLC plate using hexane/diethyl ether/acetic acid (75:25:1.5) as a mobile phase and were exposed to iodine vapors. Bands corresponding to triglycerides (TGs), diglycerides (DGs), free fatty acids (FFAs), and monoglycerides (MGs) are marked. B, Upper phases of Folch extracted basolateral media from Caco‐2 cells incubated with undigested (uBL) and digested (dBL) beef lipids, with medium only (CT) and with digested chicken lipids (dCL) were separated by thin‐layer chromatography using chloroform/methanol/water (60:35:8) as the mobile phase. TLC plates were either stained with iodine vapors (I_2_), or immunostained with the anti‐α‐Gal antibody (Ab) or with the serum of an α‐Gal allergic patient (patient's serum)

To evaluate whether α‐Gal bound to meat lipids is transported through the enterocytes, beef lipid extracts were first subjected to a simulated duodenal digestion by addition of pancreatic enzymes. For evaluation of the efficiency of digestion, thin‐layer chromatography experiments were performed with undigested lipids and with the digested lipid products that were first enriched by Folch partition. This method allows to separate the more polar lipids in the aqueous upper phase, whereas the hydrophobic lipids remain in the organic solvent‐rich lower phase. The lower phase of Folch partition containing predominantly neutral lipids, which represent the most abundant dietary lipids, was applied to the TLC plate, and hexane/diethyl ether/acetic acid was used as mobile phase (Figure 3A). A strong band corresponding to triglycerides (TGs) was only visible in the sample of undigested lipids (uBL), but was not present in the lipids that underwent digestion (dBL). Instead, a band, which most likely corresponds to free fatty acids (FFAs) released during the digestion of triglycerides, appears in the sample of the digested lipids. Furthermore, also the band corresponding to diglycerides (DGs) became more intense in the sample containing the digested lipids, where a small line corresponding to monoglycerides (MGs)^27^ appeared additionally (Figure 3A). These observations confirmed that beef lipids had indeed been hydrolyzed by the pancreatic enzymes.

As a next step, the undigested and digested beef lipids were applied to Caco‐2 cells in order to investigate the transport of α‐Gal‐binding lipids through the enterocytes. Before the actual experiments could be performed, concentrations of the digested lipids which would not affect the viability of the Caco‐2 cells had to be determined, since bile salts, needed for stabilization of lipid micelles, are cytotoxic. After overnight exposure of Caco‐2 cells to different dilutions of the micellar phases of the digested beef lipids, MTT assays were performed for evaluation of the viability of the cells. As can be seen in Figure S2, undiluted, and 1:5, 1:10 and 1:25 diluted micellar phases had a negative effect on the cells' viability, whereas dilutions of 1:50 and 1:100 did not affect the cells. Based on these results, a concentration of 1:60 was used for the following experiments.

Then, the undigested (uBL) beef lipids were added to the apical side of Caco‐2 cells and the basolateral media were collected from the lower chambers after different times of incubation (1, 2, 4 hours, and overnight). Lipids from the basolateral media were separated by Folch partition for enrichment and, since we were interested in glycolipids that might carry α‐Gal, the upper phases of Folch partition containing more polar glycolipids were applied to TLC plates. Plates were subsequently either stained with iodine (I_2_) vapors or immunostained with the anti‐α‐Gal antibody. Iodine staining of the TLC (I_2_ vapors) showed that increasing amounts of lipids were recovered from the basolateral media and applied to the plate (Figure S3). However, as can be seen in Figure S3, α‐Gal was only detected by the anti‐α‐Gal antibody after overnight incubation of the Caco‐2 cells with the lipid extract, but not after shorter exposures (1, 2, and 4 hours) of the cells to the lipids. Therefore, in all further experiments, Caco‐2 cells were incubated with the lipid extracts overnight. In a next experiment, both undigested (uBL) and digested (dBL) beef lipids as well as digested chicken lipids (dCL) and medium only (CT) were added to the apical side of Caco‐2 cells. Following overnight incubation, the basolateral media were collected from the lower chambers. After Folch partition, the obtained upper phases were applied to TLC plates, which were later either stained with iodine (I_2_) vapors or immunostained with the anti‐α‐Gal antibody or with the serum of an α‐Gal allergic individual. (Figure 3B*)*. Iodine staining (I_2_) of the TLC plates showed that comparable amounts of lipid products were obtained from the basolateral media and applied to the TLC plates. Interestingly, the anti‐α‐Gal antibody as well as patient's IgEs detected α‐Gal in the basolateral medium of cells incubated with undigested (uBL) and digested beef lipids (dBL), but not with digested chicken lipids (dCL) or medium only (CT in Figure 3B). These data showed that, regardless whether beef lipids were undigested or digested, lipids or their digestion products were transported across the Caco‐2 cells carrying still α‐Gal.

### 3.4 α‐Gal present in the basolateral medium is bound to molecules packaged into chylomicrons

**Figure 4:**
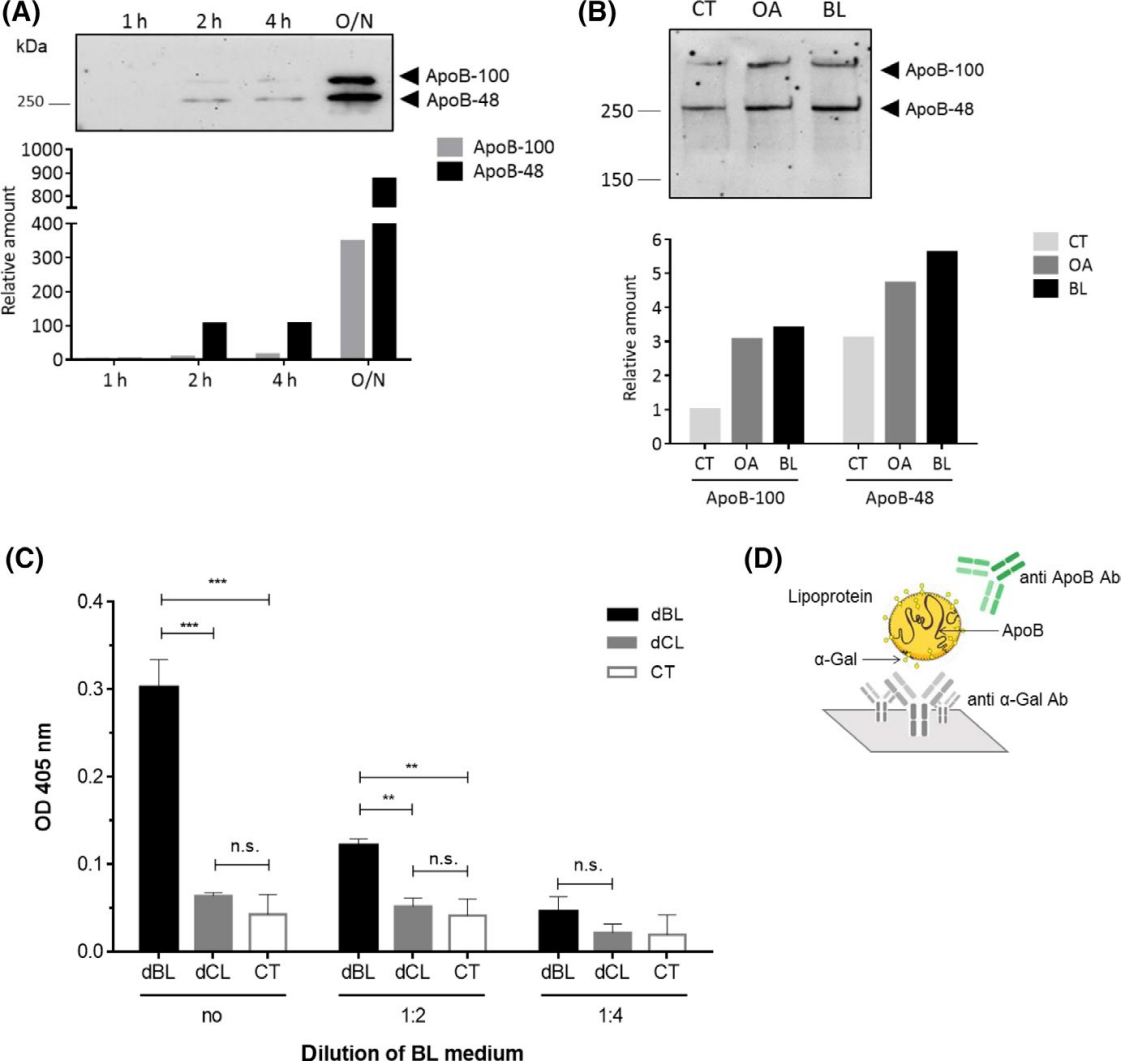
α‐Gal present in the basolateral medium is bound to chylomicrons. A, Anti‐ApoB immunoblot of basolateral media collected from Caco‐2 cells after 1 h, 2 h, 4 h, and overnight (O/N) incubation with undigested beef lipids. The graph below shows the relative amount of ApoB‐48 and ApoB‐100 at the different time points in comparison to the amount of the proteins produced after 1 h of incubation. B, Anti‐ApoB immunoblot of basolateral media collected from Caco‐2 cells incubated with only medium (CT), oleic acid (OA), and beef lipids (BL). Below, the relative amounts of ApoB‐48 and ApoB‐100 produced by the cells after being stimulated with OA and BL with respect to the amount produced by cells incubated with medium only (CT). C, Sandwich ELISA for detection of α‐Gal containing chylomicrons in the basolateral media of Caco‐2 cells that had been incubated with digested beef lipids (dBL), digested chicken lipids (dCL), or medium (CT). Media were applied either undiluted (no) or 1:2 or 1:4 diluted in PBS. In the sandwich ELISA, an anti‐α‐Gal antibody was used for catching and HRP‐labeled anti‐ApoB antibody for detection. Mean OD values indicating the binding of the anti‐ApoB antibody are shown on the *y*‐axis (**P ≤ 0.01, ***P ≤ 0.001, ns *P* > 0.05). D, Schematic representation of the ELISA set‐up

α‐Gal carried by glycolipids detected in the basolateral medium of Caco‐2 cells could be either diffusing through the cells or packaged by the enterocytes into chylomicrons. To know in which way α‐Gal glycolipids are transported, we first tested in an immunoblot whether the chylomicron marker ApoB‐48, the major protein constituent of chylomicrons, was present in the basolateral medium of Caco‐2 cells that had been incubated with lipid extracts. As can be seen in Figure 4A, where basolateral media from Caco‐2 cells that had been exposed for different times (1, 2, 4 hours, and overnight) to undigested beef lipid extracts were analyzed, an anti‐ApoB antibody recognized both apolipoprotein isoforms, ApoB‐48 (MW of 250 kDa) and ApoB‐100 (MW of 512 kDa), in the basolateral medium of the cells (Figure 4A). The expression of both apolipoprotein isoforms clearly increased over time: Whereas exposure of the Caco‐2 cells to the lipid extracts for 1 hour did not cause expression of the isoforms, exposure for 2 hours caused expression of ApoB‐48 and 4‐hours exposure led in addition also to expression of ApoB‐100. Both isoforms, especially ApoB‐48, were strongly expressed after overnight incubation of the cells (Figure 4A), indicating that chylomicrons were formed with the beef lipids. Previous studies showed that Caco‐2 cells produce chylomicrons after being stimulated with oleic acid (OA).^28^, ^29^ The amount of ApoB produced by Caco‐2 cells after incubation with beef lipids (BL) was comparable to the amount produced when they were stimulated with oleic acid (OA) (Figure 4B).

To investigate whether lipids carrying α‐Gal were packaged into these chylomicrons, a sandwich ELISA was established, in which the anti‐α‐Gal antibody was used for catching of α‐Gal containing molecules and the anti‐ApoB antibody was used for detection of the caught chylomicrons (Figure 4D). The ELISA plates coated with the anti‐α‐Gal monoclonal antibody were incubated with basolateral media collected from Caco‐2 cells incubated with digested beef lipids (dBL) or, for control purposes, with digested chicken lipids (dCL) or medium (CT, Figure 4C). Chicken lipids were again used as a negative control. The basolateral media were applied undiluted, diluted 1:2, or 1:4 in PBS. The intensity of the signal of undiluted basolateral media (no) from cells incubated with digested beef lipids (dBL) was significantly higher (*P* ≤ 0.001) than the signal of the basolateral media from cells incubated with chicken lipids (dCL) or with medium only (CT). When the basolateral media were diluted 1:2, the signal intensity was reduced. However, the signal obtained after exposure of the Caco‐2 cells to the beef lipids was still significantly higher than the background signal obtained after exposure to chicken lipids (Figure 4C). Dilution of the basolateral media of 1:4 did not result in any values above background. These experiments clearly indicated that chylomicrons produced by the enterocytes after uptake of beef lipids contained α‐Gal.

### 3.5 Basophils of an α‐Gal allergic patient are only activated by basolateral media of Caco‐2 cells exposed to α‐Gal carrying glycolipids, but not to glycoproteins

**Figure 5:**
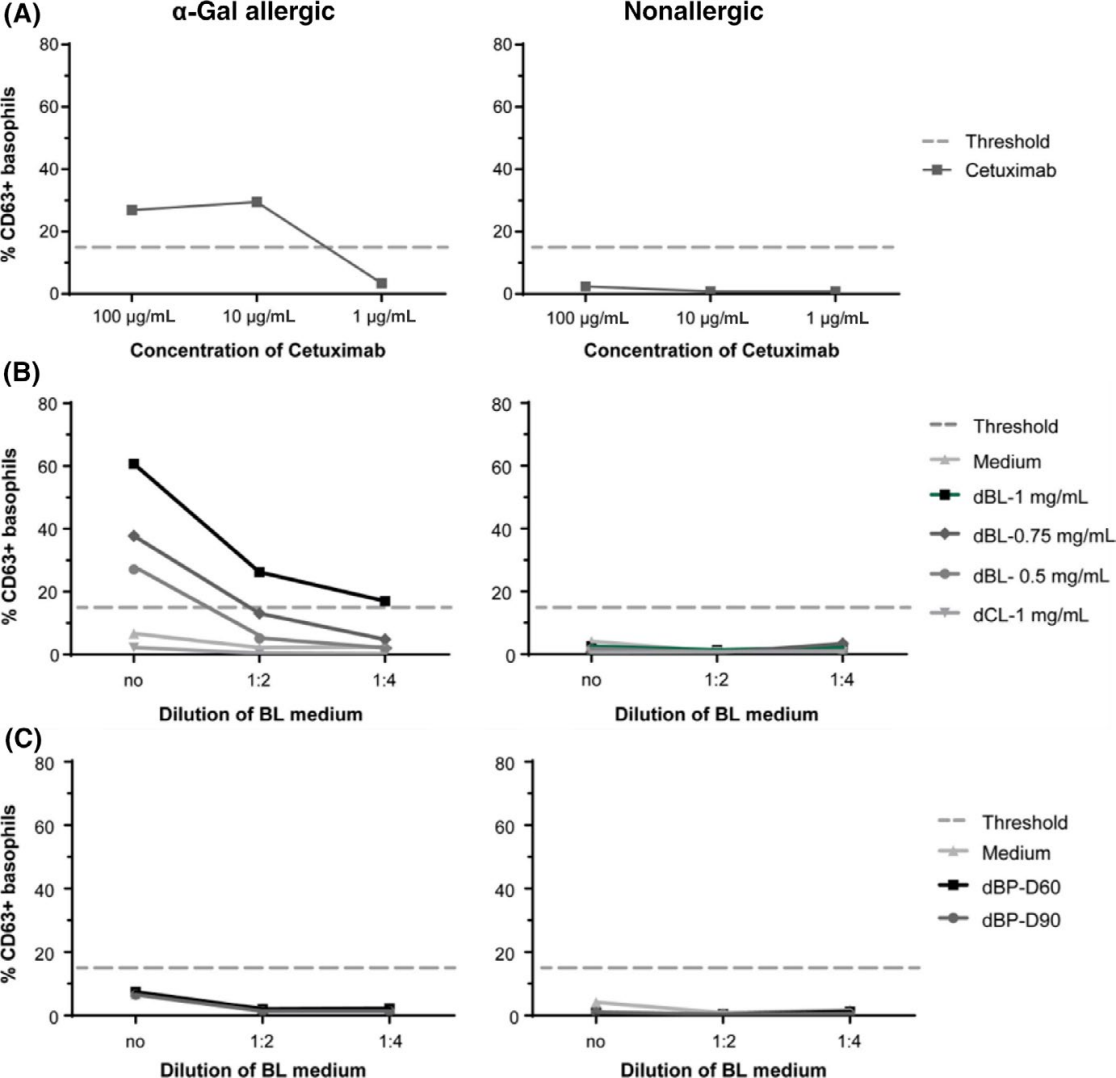
Activation of basophils form an α‐Gal allergic patient after exposure to basolateral media from Caco‐2 cells incubated with beef lipids but not with beef proteins. Basophil activation was determined by measurement of CD63 expression after incubation of whole blood of an α‐Gal allergic patient and a nonallergic donor with A, the monoclonal antibody cetuximab (in concentrations of 1 µg/mL, 10 µg/mL, and 100 µg/mL), with B, basolateral media of Caco‐2 cells exposed to 0.5 mg/mL, 0.75 mg/mL or 1 mg/mL of digested beef lipids (dBL) or 1 mg/mL of digested chicken lipids (dCL) or to medium only (always undiluted (no), or 1:2 or 1:4 diluted in PBS), with C, basolateral media of Caco‐2 cells exposed to beef proteins undergoing 60 min (dBP‐D60) or 90 min (dBP‐D90) of simulated duodenal digestion. The percentage of CD63^+^ cells is displayed on the y‐axis

A basophil activation test using blood from an α‐Gal allergic patient was performed to measure the capacity of α‐Gal moieties present on the surface of chylomicrons to cross‐link IgE antibodies and, in this way, activate effector cells. Basophil activation was determined by measurement of CD63 surface expression, where more than 15% CD63^+^ cells of the total number of basophils is regarded as positive, according to the manufacturer’s instructions (Bühlmann Laboratories AG). To assure that the basophils of the α‐Gal allergic individual could indeed be activated by α‐Gal moieties, they were first exposed to different concentrations (100, 10, and 1 µg/mL) of the α‐Gal containing monoclonal anti‐cancer antibody cetuximab.^30^ 26.9% and 29.5% of basophils from the α‐Gal allergic patient became activated with 100 and 10 µg/mL of cetuximab (Figure 5A, left panel), whereas no activation could be observed in case of the basophils from the nonallergic individual (Figure 5A, right panel). Then, the basolateral media of Caco‐2 cells incubated with different concentrations of digested beef lipids (1, 0.75, and 0.5 mg/mL), chicken lipids (1 mg/mL), or medium only were collected, and added undiluted or diluted 1:2 or 1:4 in PBS to blood cells of an α‐Gal patient and of a nonallergic individual (Figure 5B, left panel). After addition of undiluted basolateral media of Caco‐2 cells that had been incubated with 1 mg/mL digested beef lipids, 60.7% of the basophils of the α‐Gal allergic patient were activated, 37.8% of the basophils were activated with undiluted media of cells incubated with 0.75 mg/mL beef lipids, and 27.1% were activated with undiluted media of cells incubated with 0.5 mg/mL beef lipids (Figure 5B, left panel). When the basolateral media samples of Caco‐2 cells exposed to 1 mg/mL of beef lipids were diluted 1:2 and 1:4, still 26.2% and 17.0% of the basophils were activated, whereas addition of 1:2 or 1:4 diluted basolateral media of Caco‐2 cells exposed to smaller amounts of beef lipids (0.5 or 0.75 mg/mL) did not reach the 15% of positive basophils (Figure 5B, left panel). None of the tested conditions activated the basophils of the nonallergic individual (Figure 5B, right panel). These results showed that α‐Gal carrying chylomicrons present in the basolateral media were able to specifically activate basophils from an α‐Gal allergic patient. The results showed that α‐Gal carried by chylomicrons present in the basolateral media were able to specifically activate basophils from an α‐Gal allergic patient. Moreover, a higher number of basophils was activated after addition of basolateral media from Caco‐2 cells that had been exposed to higher amounts of digested beef lipids (Figure 5B, left panel), demonstrating a dose dependence in the response. In addition, more basophils became activated after stimulation with less diluted basolateral media (undiluted in comparison to 1:2 or 1:4 diluted media). Besides, basolateral medium from Caco‐2 cells exposed to chicken lipids could not activate patient's basophils (Figure 5B, left panel).

We further investigated whether exposure of Caco‐2 cells to digested beef proteins results in the transport of small α‐Gal carrying peptides through the cells. Such peptides might be too small to be detected by immunoblotting but might still have the capacity to cross‐link IgE antibodies on the surface of allergic patients' basophils. To evaluate this, Caco‐2 cells were incubated with beef proteins that had undergone 60 minutes (D60') and 90 minutes (D90') of duodenal digestion. The basolateral media were collected and added to blood cells of the α‐Gal allergic patient and the nonallergic individual. Neither proteins digested for 60 minutes (D60') nor proteins digested for 90 minutes (D90') were able to induce basophil activation (Figure 5C). The results confirmed that only when α‐Gal is carried by lipids, the oligosaccharide is transported across the enterocyte monolayer. These α‐Gal carrying lipids are then incorporated in the enterocytes into large lipoprotein structures, the chylomicrons. When chylomicrons containing α‐Gal reach the bloodstream, they can cause the cross‐linking of IgE antibodies that leads to effector cell activation.

## 4 DISCUSSION

In the present study, we used Caco‐2 cells as a model system to investigate the transport of α‐Gal carrying glycoproteins and glycolipids through intestinal epithelial cells. We saw that only α‐Gal bound to lipids, but not to proteins, was transported across the Caco‐2 cells and was able to cause the activation of basophils of an α‐Gal allergic patient. Our data have important implications for a better understanding of the phenomenon of α‐Gal allergy, and they demonstrate the striking differences between α‐Gal allergy and protein‐based food allergies.

Commins et al had shown that in α‐Gal allergy, in contrast to protein‐based food allergy, the onset of symptoms occurs 3 to 6 hours after consumption of red meat.^30^ In vitro and ex vivo basophil activation tests had further provided evidence that the reason for the delay was not a delayed response of the immune system. Instead, it had been assumed that the delay was rather caused by the way α‐Gal molecules of mammalian meat are digested and transported to the blood stream. It is known that the process of gastric and duodenal digestion and later absorption of proteins lasts for about 1 or 2 hours, from the moment they are ingested, until their amino acids are delivered to the blood.^6^, ^8^, ^31^ On the other hand, the primary postprandial peak of triglycerides in blood happens between 3 to 4 hours after starting the meal.^9^ It was therefore suggested that α‐Gal bound to lipids and their slower digestion and absorption could be behind the late responses of α‐Gal allergic patients.^14^ However, investigations on other glycolipids, like dietary sphingomyelin or plant sphingolipids, had shown that the majority of these glycolipids is hydrolyzed in the lumen of the small intestine and not absorbed and transported intact to the lymph.^32^ In contrast, it is known that some proteins, among them also allergenic proteins, as well as oligopeptides with certain hydrophobic characteristics can be transported intact through the intestinal epithelium.^15^, ^33^, ^34^ We therefore first focused on glycoproteins and investigated, whether α‐Gal was bound to such proteins that cross intact the intestinal epithelium.

In vitro digestion of beef proteins showed degradation of α‐Gal carrying proteins already after addition of pepsin (AP samples in Figure 2B). This is in accordance with previous findings.^35^ Although proteins were further degraded after addition of pancreatic enzymes, two α‐Gal carrying high molecular weight proteins were still present after 90 minutes of simulated duodenal digestion (AP‐D90 in Figure 2B). The fact that the anti‐α‐Gal antibody did not detect α‐Gal carrying proteins in any of the basolateral media collected after exposure of the Caco‐2 cells to the undigested and digested beef proteins (BL samples in Figure 2B) showed that glycoproteins were not transported through the epithelial cells carrying α‐Gal. It is expected that the majority of dietary proteins are digested by gastric and duodenal enzymes into di‐ and tripeptides or amino acids, which are then transported into the enterocytes,^36^ where they are further hydrolyzed by cytosolic peptidases. However, our Coomassie‐stained gel and our anti‐serum albumin immunoblot indicated that some beef proteins, among them serum albumin, were transported intact through the intestinal cells. These proteins not only resisted peptic and tryptic digestion, but also degradation by brush border and cytosolic peptidases expressed by Caco‐2 cells.^37^ Nevertheless, this was not the case for α‐Gal carried by proteins, since the two glycoproteins that still carried α‐Gal moieties after gastric and duodenal digestion (AP‐D90 in Figure 2B) could not be detected anymore by the anti‐α‐Gal antibody in the basolateral medium. The reason for this could either be that these proteins were degraded in the cytosol of the Caco‐2 cells or that the α‐Gal moieties were removed by enzymes present in the brush border membrane of the intestinal cells.

We then investigated whether α‐Gal bound to lipids could be transported through the intestinal cell monolayer. For this, we first proved by TLC immunostaining with an anti‐α‐Gal antibody that beef glycolipids indeed carry α‐Gal (Figure 1B). We further showed that simulated duodenal digestion resulted in hydrolysis of the lipids. When Caco‐2 cells were then exposed to undigested or digested beef lipids, α‐Gal could be detected in the basolateral media, irrespective whether the lipids underwent a previous in vitro digestion or not (Figure 3). This suggests that duodenal digestion did apparently not affect the α‐Gal moiety bound to lipids. It is known that digestion products of dietary lipids, after being taken up by enterocytes, go through a complex process where the smooth and rough endoplasmic reticulum and the Golgi apparatus collaborate to form structures called chylomicrons that are then released to the lymph vessels.^38^, ^39^ We provide evidence that polarized Caco‐2 cells, differentiated on permeable supports, mimic the intestinal epithelium and are able to form chylomicrons after exposure to meat lipid extracts. The formation of chylomicrons was proven by detection of the chylomicron marker apolipoprotein ApoB‐48 in the basolateral media (Figure 4A). Together with the truncated apolipoprotein B, ApoB‐48, also the full‐length protein, ApoB‐100, which in vivo is only synthesized in the liver as part of low density lipoproteins (LDL) and very low density lipoproteins (VLDL), was detected in the media. Previous findings showed that Caco‐2 cells cultured on porous membranes express both forms of apolipoprotein B.^40^

To investigate whether the α‐Gal bearing glycolipids of the bovine meat did not simply diffuse through the Caco‐2 cells to reach the basolateral media but were transported as part of chylomicrons, we developed a sandwich ELISA. In this ELISA, an anti‐α‐Gal specific antibody was used for catching of all α‐Gal carrying molecules and an anti‐ApoB‐48 antibody was used for detection of plate‐bound chylomicrons containing α‐Gal. Our data clearly showed that α‐Gal was bound to chylomicrons (Figure 4C). Chylomicrons are large structures of a size of about 200 nm when produced by Caco‐2 cells.^28^ However, in vivo*,* they can have sizes between 75 nm and more than 1000 nm in humans.^41^ It can be envisaged that such big structures provide large surfaces which can display many repetitive α‐Gal moieties that would be able to effective cross‐link IgE antibodies on the surface of basophils and mast cells.

We show here that the addition of basolateral media of Caco‐2 cells exposed to digested beef lipids led to activation of the basophils of an α‐Gal allergic patient (Figure 5B). On the contrary, basolateral media of cells exposed to digested proteins did not activate the patient's basophils (Figure 5C), pointing out that α‐Gal carrying peptides, too small to be detected in the immunoblots, may not be able to cross‐link IgE antibodies and trigger an allergic reaction. This demonstrates that only α‐Gal bound to lipids, but not bound to proteins, is able to cross through the enterocyte monolayer in a way that causes the activation of effector cells.

It can be speculated that the different way in which lipids are digested could lead to a higher number of unhydrolyzed, intact glycolipids carrying α‐Gal molecules. Fat droplets, emulsified in the stomach, are stabilized in the duodenum by phospholipids and bile salts, forming lipid micelles. Enzymes that participate in the digestion of lipids, such as pancreatic lipase or phospholipase, are known to work in these oil‐water interfaces.^42^ However, enzymes involved in the cleavage of oligosaccharide moieties might not be able to work at these oil‐water interfaces allowing intact α‐Gal glycolipids to enter the enterocyte.

The fact that oligosaccharides bound to lipids, but not to proteins, are able to elicit allergic reactions could explain as well the lack of allergic symptoms of patients with IgE antibodies to cross‐reactive carbohydrate determinants (CCDs) when they ingest vegetables containing CCDs, since these carbohydrates are not described to exist bound to lipids (13 and Altmann F, Vienna, Austria; personal communication). This further suggests that glycolipids represent allergenic molecules.

Furthermore, it has been observed that α‐Gal allergic individuals have, besides IgE, also elevated IgG1 levels to α‐Gal.^43^, ^44^, ^45^ This observation, together with the fact that α‐Gal reaches the bloodstream carried by chylomicrons, might explain the occurrence of bigger atheroma plaques described in patients with IgE to α‐Gal.^46^ Our findings might not only explain the late beginning of the allergic reaction in α‐Gal‐allergic individuals, due to the slower process of digestion, absorption, and release to the lymphatics of lipids, but could also associate α‐Gal with the worsening of certain cardiovascular diseases.

Our findings are supported by in vivo experiments performed in human beings, which showed that 3 to 6 hours after consumption of beef meat α‐Gal could only be detected in the blood carried by chylomicrons, but not bound to proteins (own unpublished results).

In summary, we demonstrated that only α‐Gal bound to lipids can cross the intestinal cell monolayer. In this way, it would be able to reach the bloodstream as intact oligosaccharide, which has the capacity to activate patient's basophils and trigger an allergic disease, making α‐Gal glycolipids a new allergenic molecule. Our data have important implications for a better understanding of the phenomenon of α‐Gal allergy. They demonstrate the striking differences between α‐Gal allergy and protein‐based food allergies and identify glycolipids as allergenic molecules.

## CONFLICTS OF INTEREST

The authors declare that they have no conflicts of interest.

## AUTHOR CONTRIBUTIONS

PRC designed and performed the experiments, analyzed the data, and wrote the manuscript. BL and VS contributed to the intestinal transport experiments with the Caco‐2 cells. MP and ZS were involved in the performance of the basophil activation tests. DM took part in the in vitro digestion experiments, and WH was involved in patient recruitment and participated in the interpretation of the results. IS participated in the design and planning of the experiments, in data analysis, in the interpretation of the results, and in the writing of the manuscript. All authors critically read and revised the manuscript.
