# Supplementary figures and images for: Only α‐Gal bound to lipids, but not to proteins, is transported across enterocytes as an IgE‐reactive molecule that can induce effector cell activation
Source: Allergy. 2019 Jul 16;74(10):1956–68. doi: 10.1111/all.13873 (PMC6852507; doi:10.1111/all.13873)

SUPPLEMENTARY FIGURES

Figure S1

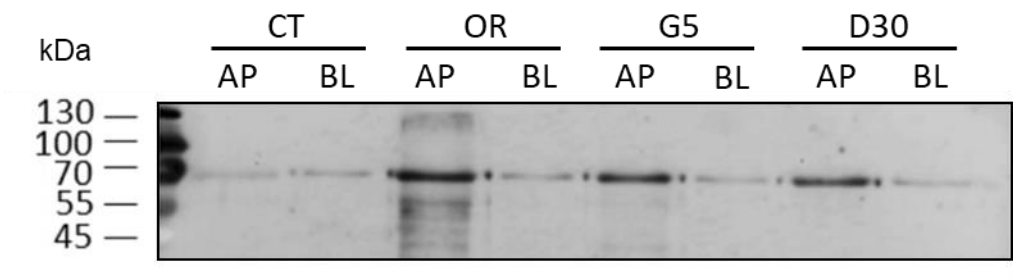

Figure S2

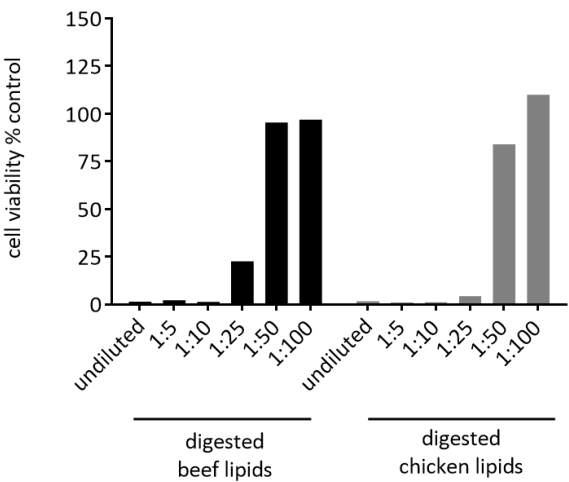

Figure S3

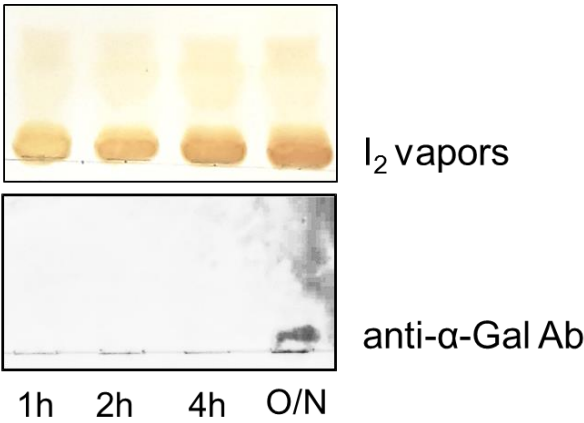

Supplement: Supplementary file 1 [file ALL-74-1956-s001.pdf]

SUPPLEMENTARY FIGURES

Figure S1

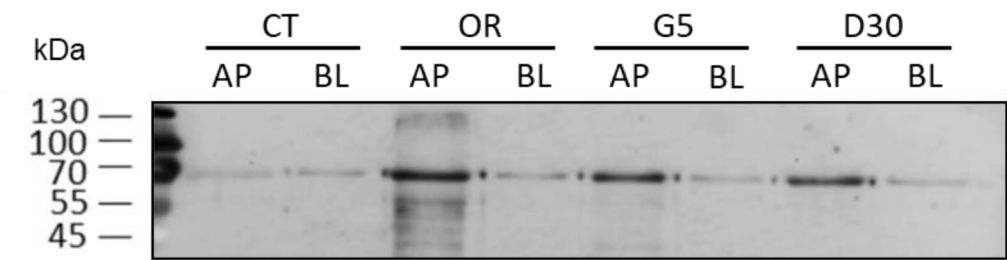

Figure S2

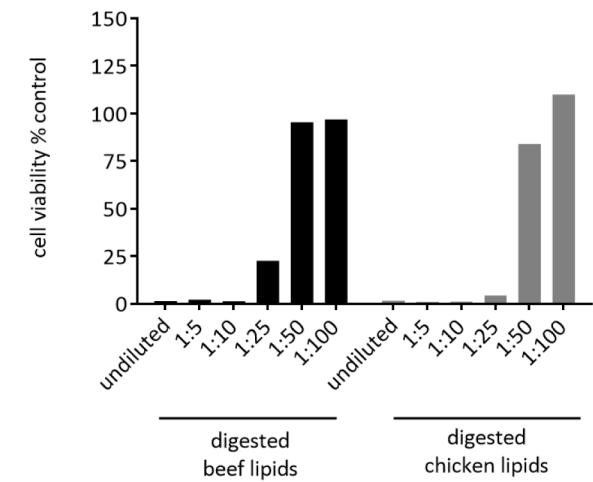

Figure S3

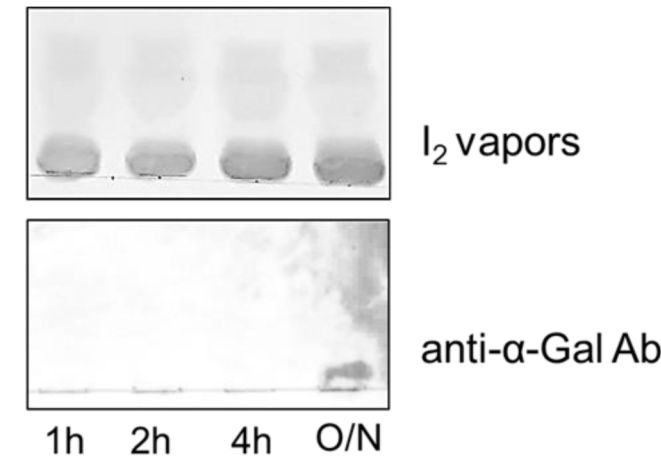

Supplement: Supplementary file 2 [file ALL-74-1956-s002.pdf]
