# Supplementary material for: Only α‐Gal bound to lipids, but not to proteins, is transported across enterocytes as an IgE‐reactive molecule that can induce effector cell activation
Source: Allergy. 2019 Jul 16;74(10):1956–68. doi: 10.1111/all.13873 (PMC6852507; doi:10.1111/all.13873)
Supplement: Supplementary file 3 [file ALL-74-1956-s003.docx]

## SUPPLEMENTARY MATERIAL AND METHODS

### Patient

Whole blood and serum were obtained from a 39-year-old, male farmer. The patient experienced generalized urticaria, shortness of breath, weakness, dizziness, impaired vision, tachycardia and hypotension 3-4 h after consumption of sausages followed by heavy labor, and he needed emergency treatment and subsequent hospitalization. The patient’s history revealed that he had been suffering progressively from urticaria attacks associated with breathing difficulties, dizziness and tachycardia after consumption of pork, beef, lamb, and various sausages for more than 10 years. No symptoms occurred after consumption of poultry meat, fish or seafood. Total IgE (639 kU/l) and specific IgE levels to pork (7.42 kUA/l), beef (8.30 kUA/l) and α-Gal (287 kUA/l) were determined by ImmunoCAP® (Thermo Fisher, Uppsala, Sweden).

The study was approved by the Ethics Committee of the City of Vienna (EK-12-126-0712) and the patient gave written informed consent to participate in the study.

### SDS-PAGE and immunoblot

For evaluation of the presence of α-Gal on undigested beef and chicken glycoproteins, extracts were separated by 12% SDS-PAGE (Laemmli 1970) and gels were either stained with Coomassie Brilliant Blue or blotted onto nitrocellulose membranes. For detection of α-Gal, membranes were blocked with 0.1% HSA in PBS-T (PBS containing 0.5% Tween-20) and then incubated with a HRP-labeled anti-α-Gal antibody (m86 mAb, Absolute Antibody) diluted 1:1000 in PBS-T. Antibody binding was detected with a chemiluminescent HRP substrate (SuperSignal®West Pico, Thermo Fisher Scientific) using the FluorChem®, Protein simple device (Biozyme Scientific GmbH, Hessisch-Oldendorf, Germany). For analysis of digested protein extracts, which were applied to the apical side of Caco-2 cells, and of basolateral media collected after incubation of Caco-2 cells with protein extracts, digested extracts (AP) and basolateral media (BL) were separated by 16.5% Tris-Tricine polyacrylamide gel electrophoresis (PAGE) and either stained with Coomassie Brilliant Blue or blotted onto nitrocellulose membranes. α-Gal was detected as described above. For detection of bovine serum albumin (BSA) in the digested extracts and in the basolateral media, they were separated by 12% SDS-PAGE followed by blotting onto nitrocellulose. Membranes were incubated with an anti-serum albumin antibody (ab112991, Abcam, Cambridge, UK) diluted 1:10,000 in PBS-T and a HRP-labeled donkey anti-goat IgG secondary antibody (ab97110, Abcam, Cambridge, UK) diluted 1:1,000,000 in PBS-T. Antibody binding was detected as described above.

Production of chylomicrons by Caco-2 cells incubated with beef lipid extracts was evaluated by detection of apolipoprotein B-48 (ApoB-48) in the basolateral medium by immunoblotting. Medium from the basolateral chambers was separated by 6% SDS-PAGE, followed by blotting onto nitrocellulose membranes. After blocking with 0.1% HSA in PBS-T membranes were incubated with the anti-ApoB mAb (clone 7B8, Thermo Fisher Scientific) diluted 1:5000 in PBS-T. After incubation with the secondary antibody (HRP-labeled anti-mouse IgG antibody; diluted 1:2000 in PBS-T), the detection of reactive bands was carried out with the chemiluminescent HRP substrate (SuperSignal®West Pico, Thermo scientific) on the FluorChem®, Protein simple device. The intensities of signals of ApoB-48 and ApoB-100 were analyzed using ImageJ (National Institutes of Health, Bethesda, MD). Data were reported as relative amounts. The amount of ApoB-48 and ApoB-100 produced after 1 h of incubation were used as the reference to which the signals obtained after longer incubation were normalized.

### Culture of Caco-2 cells

Human epithelial colorectal adenocarcinoma cells, Caco-2 (300137, CLS GmbH, Eppelheim, Germany) were cultured in T-175 flasks in high-glucose (25 mM) Dulbecco’s modified Eagle’s medium (DMEM) (Sigma) supplemented with 10% fetal bovine serum (FBS, Gibco™ Thermo Fisher Scientific, Waltham, MA), 2% L-glutamine and 1% Penicillin-Streptomycin (10% FBS high-glucose DMEM) in a humidified atmosphere with 5% CO_2_ at 37°C as described(47). Once the cells reached 80-90% confluence, the cells were passaged and seeded on PET membranes of ThinCert™ tissue culture inserts (1 µm pore size, 24 mm diameter, Greiner Bio-One, Kremsmünster, Austria) at a density of 4.3×10^5^ cells/insert. After 8 days in 10% FBS high-glucose DMEM cells were grown for 2 more weeks in low-glucose (5mM) DMEM under asymmetric culturing conditions: medium containing 10% FBS was applied only to the lower compartment, while FBS-free medium was added to the upper chamber. Media were replaced every second day and cells were used for the transport experiments after 21 days, when a tight monolayer of fully differentiated cells was formed. The formation of tight monolayers was assessed by measuring the transepithelial electrical resistance (TEER) before and after application of the protein and lipid extracts using the EVOM resistance meter (World Precision Instruments, Sarasota, FL).

### Cell viability assay

Potential negative effects of the digested lipids on cell viability were evaluated using the MTT viability assay in a 96-well format. Due to the cytotoxic effects of high concentrations of bile salts, which are necessary to stabilize the lipid micelles, the viability of Caco-2 cells was evaluated after addition of different concentrations of the micellar phases from lipid extract digestions.

For the MTT assay, Caco-2 cells were incubated overnight with micellar phases of digested beef and chicken lipids undiluted or diluted 1:5, 1:10, 1:25, 1:50, 1:100 (v/v) in low-glucose DMEM. Cells were washed with PBS and 100 µl of MTT working solution, containing a final concentration of 0.83 mg/mL of 3-(4,5-Dimethylthiazol-2-yl)-2,5-diphenyltetrazoliumbromide (MTT) diluted in FBS-free medium (1:5) was added to the wells. MTT working solution was removed after 15 min and the purple formazan formed during incubation was dissolved in 150 µL DMSO per well. Absorbance was then measured using a multi-well plate reader (Tecan infinite M200, Tecan, Grödig, Austria), at 570 nm with 650 nm as reference wavelength. The cell viability was assessed relative to untreated control cells (Fig E2).

### Basophil activation test

Basophil activation tests (BATs) were carried out according to the manufacturer’s instructions (Bühlmann Laboratories AG, Schönenbuch, Switzerland). Whole blood was collected from the α-Gal-allergic patient and from a nonallergic donor in EDTA tubes and was used within 2h after drawing. BATs were performed with various concentrations of cetuximab (Erbitux®) (Merck, Vienna, Austria) diluted in PBS (100 µg/ml; 10 µg/ml and 1 µg/ml) and with the basolateral media from Caco-2 cells that had been exposed to the following samples: to the micellar phases of beef lipids digested at concentrations of 0.5 mg/ml, 0.75 mg/ml and 1 mg/ml; to the micellar phases of chicken lipids digested at a concentration of 1 mg/ml; to aliquots of the digested beef proteins extracts (60 min duodenal digestion, 90 min duodenal digestion). The basolateral media were either applied undiluted or 1:2 and 1:4 diluted in PBS. The basolateral medium from cells incubated with lipids was first centrifuged at 13,000xg for 20 min to enrich chylomicrons(22). Due to their low density the chylomicrons floated on top of the solution. They were recovered and further diluted with PBS as described.

In addition to the samples mentioned above, basophils were also stimulated with two positive controls, namely fMLP (formyl-methionyl-leucyl-phenylalanine), an anti-FcԑRI antibody (Bühlmann Laboratories AG, Schönenbuch, Switzerland) and with stimulation buffer as negative control. Basophils were always incubated at 37°C for 25 min and stained for expression of CCR3 and CD63 (Bühlmann Laboratories AG, Schönenbuch, Switzerland). Basophils were gated as low side‐scattered CCR3^+^ cells and their activation was determined by an increased surface expression of CD63. The measurements were performed in a FACSCanto II cytometer (BD Biosciences, San Jose, CA). A minimum of 500 basophils were acquired and analyzed with FACSDiva™ software (BD Biosciences, San Jose, CA)**.**
